# Massive pleural effusion following high-power and short-duration radiofrequency ablation for treatment of atrial fibrillation: A case report and review of the literature

**DOI:** 10.3389/fcvm.2022.996481

**Published:** 2022-10-14

**Authors:** Miaomiao He, Jie Qiu, Yang Bai, Yan Wang, Guangzhi Chen

**Affiliations:** Division of Cardiology, Department of Internal Medicine, Tongji Hospital, Tongji Medical College, Huazhong University of Science and Technology, Wuhan, China

**Keywords:** atrial fibrillation, radiofrequency catheter ablation, postpericardial injury syndrome, diagnosis, pleural effusion

## Abstract

Postpericardial injury syndrome (PPIS) is defined as pericarditis or pericardial effusion that results from recent myocardial infarction or intracardiac interventions. These symptoms typically include fever, leukocytosis, a high erythrocyte sedimentation rate, and elevated C-reactive protein levels. Additionally, pericardial effusion and pleural effusion may be present. It is considered to be a common complication in cardio-surgery with an occurrence of 3–30%. In the past 20 years, a high number of patients with atrial fibrillation have suffered from PPIS following radiofrequency catheter ablation. However, previous reports focused on identifying cardiac tamponade and pericardial effusion as their main clinical manifestations. Solitary pulmonary involvement following PPIS with the radiofrequency catheter ablation may occur. We report a case of PPIS that presented pleural effusion as the dominant feature soon after the operation and systematic review to illustrate the clinical characteristics of PPIS.

## Introduction

Radiofrequency catheter ablation (RFCA) involves pulmonary vein isolation and left atrial ablations, which are a crucial part of non-pharmacological treatment for drug-refractory atrial fibrillation (AF) (1–3). RFCA has become more widely used in the treatment of uncontrolled AF in the past few years (4, 5). As a result of RFCA, complications such as left atrial esophageal fistula and cardiac tamponade have declined over the past 10 years, especially when performed by an experienced surgeon (6, 7).

Postpericardial injury syndrome (PPIS) is defined as pericarditis or pericardial effusion that results from myocardial infarction or intracardiac interventions (8). These symptoms typically include fever, leukocytosis, a high erythrocyte sedimentation rate, and elevated C-reactive protein levels. Additionally, pericardial effusion and pleural effusion may be present. The first described PPIS for cardiac surgeries was reported in 1958 (9). It is considered to be a common complication in cardio-surgery with an occurrence of 3–30% (10, 11). PPIS following RFCA of AF has been frequently reported over the past 20 years. But the majority of reported cases concerning PPIS focused on simultaneous pleural and pericardial effusion as first clinical manifestations. There is no reported case of solitary pulmonary involvement except for a new case from our center. Hence, considering the challenging nature of this disease, we here present an unusual case of PPIS manifested by massive pleural effusions alone and a systematic review to illustrate clinical characteristics of PPIS.

## Case presentation

**Figure 1:**
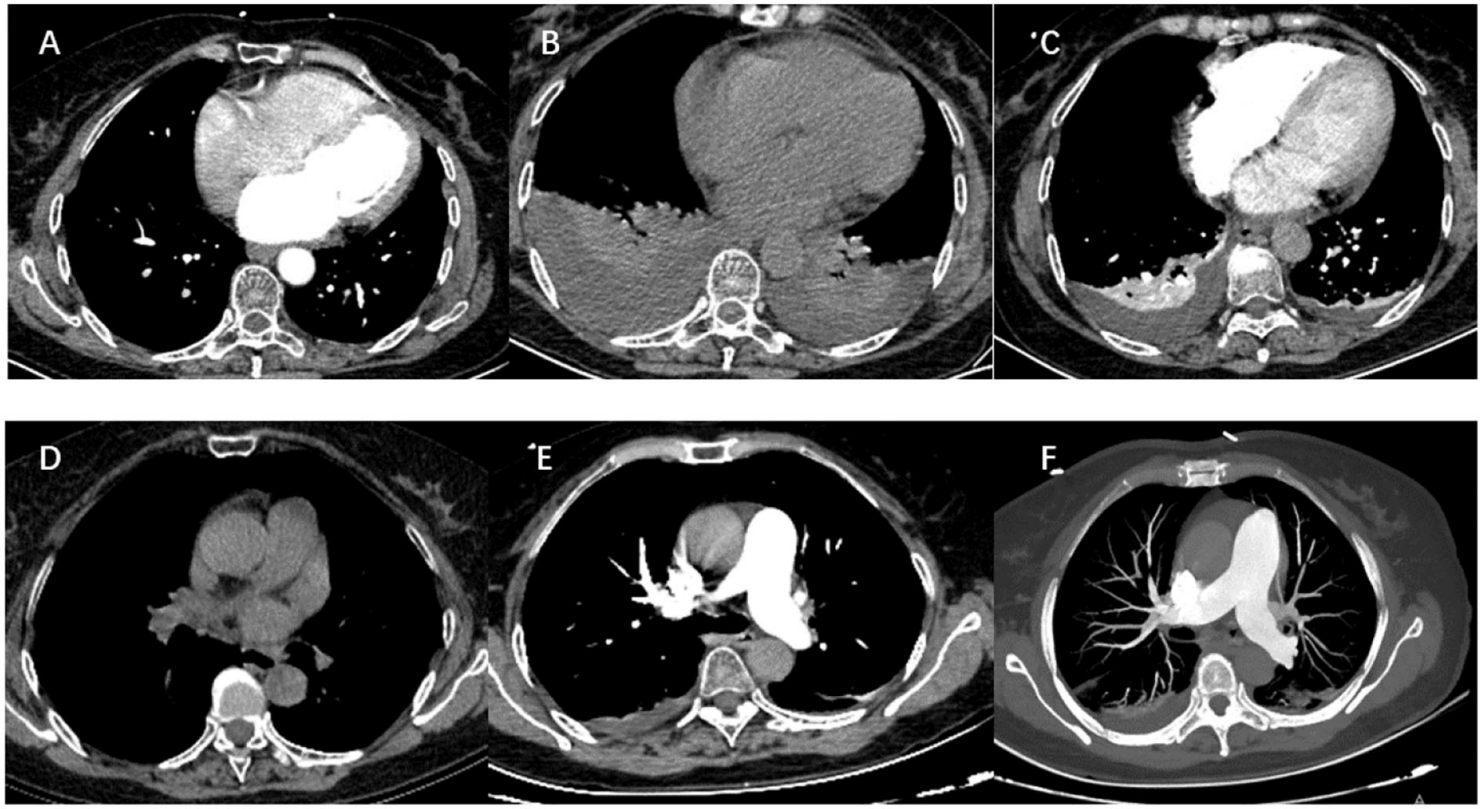
The dramatic transition of a thoracic CT scan (soft-tissue window) and pulmonary computed tomography angiography. **(A)** Preoperative tests: CT revealed clear lung fields bilaterally. **(B)** Three days after operation: CT scan showing large bilateral pleural effusions without pericardial effusion. **(C)** Nine days after the operation: there is a resolution of the left pleural effusion and a marked decrease in the right pleural effusion. **(D)** Nineteen days after operation: there is no pleural effusion on CT images. **(E,F)** Pulmonary computed tomography angiography ruled out pulmonary embolism.

A 65-year-old woman underwent CA at our center because of increasing palpitation symptoms despite antiarrhythmic drug therapy. She had a history of hypertension, and chronic AF and had symptomatic AF confirmed by the 12-lead ECG for 1 year (**Figure 3A**). On admission, her routine clinical assessment and physical examination revealed irregular heart sounds, jugular venous pulsations, and hypertension. Preprocedural transesophageal echocardiography showed normal biventricular function with patent foramen ovale and no thrombus in the LA appendage. A chest computed tomography demonstrated no significant abnormalities, as shown in Figure 1A.

**Figure 2:**
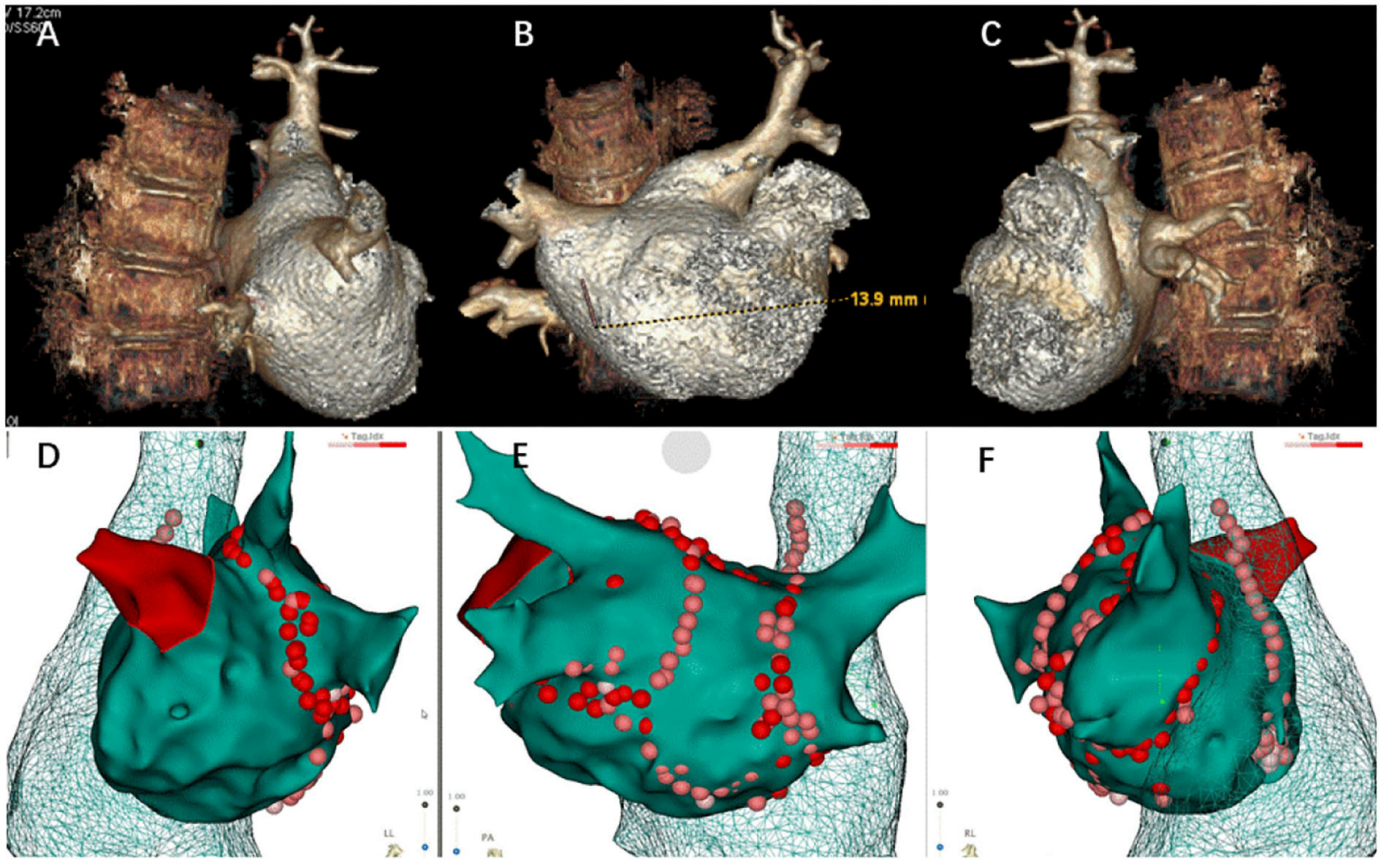
Three-dimensional reconstruction of pulmonary vein and ablation circle (red-colored) using the CARTO3^®^ system. **(A)** Three-dimensional reconstruction of the pulmonary vein in left lateral view. **(B)** Three-dimensional reconstruction of the pulmonary vein in posteroanterior view. **(C)** Three-dimensional reconstruction of the pulmonary vein in right lateral view. **(D)** Ablation circle in left lateral view. **(E)** Ablation circle in posteroanterior view. **(F)** Ablation circle in right lateral view.

After a successful routine single transseptal puncture, under electroanatomic mapping data using a 3D mapping system (CARTO3, Biosense Webster, Inc, Diamond Bar, CA), all four pulmonary veins were isolated and the additional ablation was technically successful using a ThermoCool SmartTouch irrigation-tip contact force radiofrequency ablation catheter (Biosense Webster Inc, Irvine, CA), including groof of the left atrium, BOX isolation of posterior wall, superior vena cava, and right atrial cavotricuspid isthmus. Depending on the ablation index, ablation was initiated at a power of 60 W for a duration of 8–10 s on the left posterior wall and 11–20 s on the other parts of the left atrial wall. We ablated the cavotricuspid isthmus and other parts of the right atrial wall with 40 W and adjusted the target ablation index between 400 and 500 if needed. The superior vena cava was ablated with 40 W and the target ablation index was adjusted between 250 and 350 as necessary. To avoid excessive drops in impedance, we adjusted the contact force by 5–10 *g* for each application (Figure 2). A total of 7,000 U heparin was given during the procedure. The scheduled procedure was completed without complications. The vital signs were stable during ablation.

**Figure 3:**
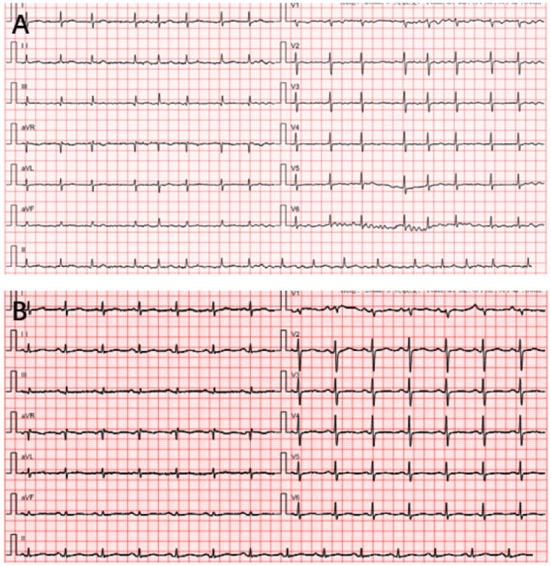
Patient's electrocardiogram (ECG). **(A)** The preprocedure ECG reveals atrial fibrillation. **(B)** The postoperative ECG of the patient when she felt palpitations and chest discomfort.

On the following day, there was a progressive worsening of symptoms associated with chest distress and dyspnea (Figure 3B). Upon physical examination, her neck veins were non-distended, her lungs were distant, and her heart sounds were clear. She was afebrile, with a normal sinus rhythm of 120 breaths per minute, blood pressure of 135/76 mmHg, and respiratory rate of 20 breaths per minute. There was a mild rise in the inflammatory markers (C-reactive protein [CRP]). High sensitivity cardiac troponin T was elevated at 2,546.1 pg/ml (Table 1). Significant laboratory findings included a white blood cell count of 16.82 × 10^9^/L with 87.4% neutrophils. A transthoracic echocardiogram (TTE) showed normal left ventricular function (ejection fraction 60%) without pericardial effusion, but a new-onset small right pleural effusion was detected. An oral diuretic was prescribed, which improved the symptoms. The patient was started on glucocorticoids, antibiotic therapy, and oxygen inhalation by mask.

**Table 1:** Several laboratory data cases showing the change in leukocytosis and vital sign.

	**WBC** **(10^9^/L)**	**Hb** **(g/L)**	**NT-proBNP** **(pg/mL)**	**EF** **(%)**	**T (°C)**	**HR** **(Times / minute)**	**R** **(Times / minute)**
1 week before operation	8.77	130	732	68	36.3	105	20
1 day after operation	16.82	116	217	60	36	57	20
3 days after operation	16.26	117	173	64	36.6	61	21
1 week after operation	18.1	118	113	67	36.3	72	19

WBC, white blood cell; Hb, hemoglobin; NT-proBNP, N-terminal pro-B-type natriuretic peptide; EF, ejection fraction; T, temperature; HR, heart rate; R, respiratory rate.

Her health condition did not improve after treatment for 3 days. As shown in Figure 1B, massive bilateral pleural effusion alone was observed in a chest computed tomography. There was no pericardial effusion in the post-procedure TTE. Pulmonary computed tomography angiography ruled out pulmonary embolism (Figure 1). After the exclusion of infectious, metabolic, and toxic causes of pleural effusion, the criteria for PPIS were considered because our patient was found to have pleural effusions along with pleuritic chest distress and elevated levels of inflammatory markers. Then the patient was continued on glucocorticoid and antibiotic therapy. The pathological changes were resolved and the chest CT reverted to normal after 3 days of treatment, and the patient was able to be discharged out of the hospital; the chest CT is shown in Figure 1C.

At a 1-month follow-up, recovery of the patient was uneventful, and the chest CT scan displayed full expansion of the lungs with almost complete resolution of the massive pleural effusion (Figure 1D).

## Literature review

### Methods

A systematic electronic literature search for primary evidence was performed in the PubMed database. Keywords used in electronic searching include “post-cardiotomy syndrome”, or “post-cardiac injury syndrome”, or “Dressler's syndrome,” or “pericardial effusion, and CA.” No language restrictions were applied. In addition to the articles searched by keywords, the reference lists of all relevant articles were also examined. Articles satisfying the following criteria were included in this study: (1) the main clinical manifestation after the operation is pleural effusion and (2) CA treatment in patients with AF. Studies that meet the following criteria were excluded: (1) studies not in the English language; (2) studies published only in abstract or review form; and (3) data unavailable, or not relevant. Other ablation-related complications such as cardiac tamponade, left atrial esophageal fistula, pericarditis, and pericardial effusion were not enrolled in this study.

### Results

Based on the key terms used for the search, 561 articles were initially identified between 1993 and 2022. We excluded 33 non-English studies during the screening of abstracts; 524 articles that did not meet the inclusion criteria were also ruled out. After screening titles and corresponding contents, 11 published studies were identified as fulfilling the inclusion criteria. The extracted data included the name of the author, year of publication, age and gender of patients, type of AF, the onset of catheter-related complications, laboratory examination, outcomes, and therapeutic strategies. The extracted data are compiled in Table 2.

**Table 2:** Clinical characteristics of all cases reported in the literature.

**Reference**	**Basic information**					**Clinical manifestation**															**Elevated CRP/ESR**	**Elevated WBC**	**Treatment**						**Follow-up**
	**Age**	**Sex**	**Type of AF**	**Watt of AF**	**Time of PPIS**	**Vomiting**	**Dyspnea**	**Fever**	**Chest pain**	**Cough**	**Pleural rub**	**Pulmonary infiltrates**	**Pneumonia**	**Pleural effusion**			**Pericardial rub**	**Pericardial effusion**	**Cardiac perforation**	**Cardiac Tamponade**			**NSAID**	**Steroids**	**Colchicine**	**Antibiotic therapy**	**Pericardiocentesis**	**Thoracentesis**	
														**Bilateral**	**Unilateral (side)**	**Time of detection**													
Wood et al. (12)	54	M	persistent AF	12W	5 DAYS	NO	YES	YES	YES	NO	NO	NO	NO	YES	NO	5DAYS	NO	YES	NA	YES	YES	NA	YES	NO	NO	YES	YES	YES	3 MON
Luckie et al. (13)	56	M	AF	NA	8 WEEKS	NO	YES	YES	YES	NO	NO	NO	NO	NO	NO	~	YES	MODERATE	NO	NO	YES	NO	YES	YES	YES	NO	NO	NO	6 WEEKS
Goossens et al. (14)	68	F	paroxysmal AF	25-35W	4 DAYS	NO	YES	YES	YES	NA	NA	NO	NO	YES	NO	2 WEEKS	NO	YES	NO	YES	YES	NA	YES	YES	NO	NO	YES	YES	NA
Liu et al. (15)	60	F	paroxysmal AF	35W	2 DAYS	NO	YES	YES	NO	NO	NO	NO	YES	YES	NO	2 DAYS	NO	YES	YES	NO	YES	YES	YES	NO	NO	NO	YES	NO	1 MON
Liu et al. (15)	77	F	paroxysmal AF	35W	3 DAYS	NO	YES	YES	NO	NO	NO	YES	YES	YES	NO	3 DAYS	NO	YES	YES	NO	YES	YES	NO	YES	NO	YES	YES	NO	1 MON
Liu et al. (15)	56	M	paroxysmal AF	35W	2 DAYS	YES	NO	YES	YES	NO	NO	YES	YES	NO	YES (left)	2 DAYS	NO	YES	YES	NO	NO	NO	YES	NO	NO	NO	NO	NO	1 MON
Liu et al. (15)	67	M	persistent AF	35W	4 DAYS	NO	YES	NO	YES	NO	YES	YES	NO	YES	NO	4 DAYS	NO	YES	YES	NO	NO	NO	NO	NO	NO	NO	NO	NO	1 MON
Liu et al. (15)	62	F	paroxysmal AF	35W	3H	NO	NO	NO	YES	NO	YES	NO	NO	NO	YES (left)	3H	NO	YES	YES	NO	NO	NO	NO	NO	NO	NO	YES	NO	1 MON
Torihashi et al. (16)	49	M	persistent AF	25-30W	2 WEEK	NO	NO	NO	YES	NO	NO	NO	NO	NO	NO	~	YES	LARGE	NO	YES	YES	YES	YES	NO	NO	NO	YES	NO	4 DAY
Yukumi et al. (17)	24	M	paroxysmal AF	NA	3 MONTHS	NO	NO	YES	YES	NO	NO	NO	NO	NO	YES (right)	3 MONTHS	NO	YES	NO	NO	YES	YES	YES	NO	YES	NO	NO	YES	1 MON
Han et al. (18)	68	F	paroxysmal AF	30W	1 DAY	NO	YES	YES	YES	YES	NO	YES	YES	NO	NO	~	NO	NO	NO	NO	YES	YES	NO	YES	NO	YES	NO	NO	1 MON
Li et al. (19)	82	M	persistent AF	NA	14 DAYS	NO	NO	YES	NO	NO	NA	YES	NO	YES	NO	14 DAYS	NO	YES	NO	NO	YES	NO	NO	YES	NO	YES	NO	YES	1 MON
Li et al. (19)	78	M	persistent AF	NA	3 DAYS	NO	YES	YES	NO	YES	NA	MASSIVE	NO	YES	NO	3 DAYS	NO	SMALL	NO	NO	YES	YES	NO	YES	NO	YES	NO	NO	1 MON
Fong et al. (20)	59	F	paroxysmal AF	NA	3 WEEK	NO	NO	YES	NO	YES	NO	NO	NO	NO	NO	~	NO	LARGE	NO	NO	YES	YES	NO	YES	NO	NO	YES	NO	2 WEEK
Rosati et al. (21)	62	F	persistent AF	35W	2 WEEK	NO	YES	YES	YES	YES	NA	NO	NO	NO	YES (left)	1 MON	NO	MODERATE	NA	NO	NA	YES	YES	NO	YES	NO	YES	YES	2 WEEK
Fukasawa et al. (22)	69	M	paroxysmal AF	35-40W	1 MON	NO	YES	NO	YES	NO	NA	NO	NO	YES	NO	3 MONTHS	NA	YES	NO	YES	YES	NA	NO	NO	YES	NO	YES	YES	24 MON
This study	65	F	persistent AF	60W	3 DAYS	NO	YES	NO	YES	NO	NO	NO	NO	YES	NO	3 DAYS	NO	SAMLL	NO	NO	YES	YES	NO	YES	NO	YES	NO	NO	2 WEEK

F, female; M, male; W, watt; Mon, month; AF, atrial fibrillation; PPIS, post-pericardiotomy syndrome; CRP, C-reactive protein; ESR, erythrocyte sedimentation rate; WBC, white blood cell; NSAID, Nonsteroidal anti-inflammatory drugs.

Data of 17 diagnosed patients with PPIS including 16 (94.1%) from the 11 articles and one new case from our center were collected. There were eight female and nine male patients with a mean age of 62.1 years (range: 24–82 years). Of the 17 patients, nine had paroxysmal AF and seven had non-paroxysmal AF. The presence of symptoms associated with PPIS usually initiated within 3 h to 3 months after RFCA, with an average of 15 days after ablation. The predominant symptoms included pleural effusion and pericardial effusion, which mostly occurred in the first week (52.9%, 9/17). Most patients had dyspnea (11/17, 64.7%), chest pain (12/17, 70.5%), and fever (12/17, 70.5%). Interestingly, 12 cases with pleural effusion presented pericardial effusion except for a new case. Another specific clinical sign is that low power delivery (20–40 W) over a long duration (20–40 s) was performed in the majority of cases. Elevated markers of inflammation and elevated WBC are also important clinical signs and were present in 76.4% of cases (13/17) and 52.9% of cases (9/17), respectively. In one case, the appearance of resistant ascites and progressive prominent symptoms of congestion pointing to the diagnosis of constrictive pericarditis arose 1 month after the ablation of AF (22). Therapeutic strategies for pleural effusion following ablation of AF were based on nonsteroidal anti-inflammatory drugs (NSAIDS) (8/17, 47.1%), glucocorticoids (8/17, 47.1%), and antibiotic therapy (6/17, 35.2%). Pericardiocentesis was presented in 9 cases, and thoracentesis was presented in 6 cases. No mortality occurred during a mean follow-up of 2.3 (0.5–24) months.

## Discussion

In this study, we present the first case of PPIS characterized as massive pleural effusion alone after RFCA of AF. In our case, a symptom cluster of chest tightness and breathlessness occurred the day after the operation. Contrast radiography suggested massive bilateral pleural effusion in the absence of pericardial effusion. Cardiac tamponade and cardiac perforation were ruled out by echocardiography. Pulmonary computed tomography angiography also ruled out pulmonary embolism (Figure 1). PPIS after the operation was considered when both elevated leukocyte count and increased C-reactive protein. RFCA is currently the most commonly used ablation technique for the treatment of AF, aiming at eliminating AF and maintaining sinus rhythm in long term. Conventional thermal radiofrequency ablation for AF is low power delivery (20–40 W) over a long duration (20–40 s). Recently, there has been increasing interest to use relatively higher power (45–70 W) over a short duration (5–10 s) (23). The goal was to achieve a high rate of transmural trauma with minimal destruction of surrounding tissues, resulting in lower rates of recurrence and higher efficiency of this solution. Compared with conventional radiofrequency ablation, a large number of studies confirmed that HPSD may create transmural lesions but lessen injurious heating of deeper structures (24–26). This particular patient was successfully treated with 60 W RFCA. The sinus rhythm of the patient recovered following the completion of pulmonary vein isolation and did not complain any discomfort, which suggested that the operation process was smooth and safe.

Most likely, the primary cause of PPIS is an autoimmune phenomenon, but the precise mechanism remains unknown (27). There are two theories to explain the origin of this syndrome. The first theory is that antibodies against contractile proteins actin and myosin (AMA) and circulate immunocomplex were produced after surgical trauma, resulting in the exposure of endogenous antigens (28, 29). It has been reported that the epicardium after myocardial injury contributed to an inflammatory response by generating cytokines, which led to modulate revascularization and repair of damaged tissue and incite an inflammatory reaction (30). Some studies have confirmed that the presence of specific auto-antibodies experienced a 4-fold increase in the postoperative period, which provides evidence supporting the autoimmune etiology. The second theory is that autoimmune reaction is accelerated by a recent or reactivated viral infection (31). Concomitant mechanical injury of the pericardium is necessary for both theories. In the articles included in the present systematic review, the case in which clinical signs included massive pleural effusion was performed with the application of postoperative thoracentesis and thoracostomy tube placement. Although the patients in our case presented with massive pleural effusion alone, the treatment of chest drain insertion was not given. One reason is that the patient's vital signs were stable, and her oxygen saturation was above 95% under oxygen inhalation. Another is that the patient had a better response to therapy. Within 3 days of treatment with glucocorticoids, antibiotic therapy, and NSAIDS, the patient showed a gradual decrease in the large volumes of pleural fluid, and thoracic CT became normal at recheck examination 2 weeks after discharge. On-demand use of NSAIDs and glucocorticoids, a rapid symptomatic improvement in the case presented strongly argues for an immune-mediated mechanism.

A comprehensive systematic review of PPIS has shown that there was a predominance of CA of AF to ablation-associated PPIS (71.4%) (19). This reflects the fact that AF ablation was associated with a higher risk of PPIS than other RFCA procedures because of larger defects in the myocardium and a higher probability of injury to the adjacent vessels and pleura (15, 32). It has been reported that the incidence of PPIS is correlated to the extent and progression of myocardial damage.

The incidence of PPIS is greater in procedures that involve an extensive area of the myocardium. As is well known, CA of AF usually causes extensive linear lesions of the atrial myocardium, particularly following persistent AF ablation (33). These may have caused the increased incidence of PPIS. However, with all the recent advances in techniques of zero X-ray ablation approach, CF-sensing catheters, and ablation index (34–36), an attempt is being made to reduce the incidence of complications of tamponade and radiation. In the smart AF trial, the incidence of cardiac tamponade was 2.5% among 161 patients (37). In the Toccastar Trial, the tamponade incidence was much lower (38). The results of one recent clinical trial show that the use of AI was associated with a lower observed rate of tamponade (36).

Petey et al. reported a similar case to ours in 2008. Their case was also simple, with exudative pleural effusion following pulmonary vein isolation for paroxysmal AF. However, their case developed PPIS after the thoracoscopic procedure, which is different from our case. In 2013, Yang Liu et al. published an article on the main complications associated with RFCA of cardiac arrhythmia, especially the incidence of PPIS. Only 6 cases of PPIS have been reported in the literature, of whom five were involved in CA of AF. This is because AF ablation carries a higher risk of cardiac perforation than other RFCA procedures. These patients became symptomatic of dyspnea, fever, pleural effusion, or pericardial effusion. Pericardial effusion was present in three of the four patients who had pleural effusions. In a notable departure from a paper published in 2013, radiofrequency ablation AF by use of the Carto3 system was performed in our case rather than the EnSite system. The relatively higher power (60 W) over a short duration was used in our case, rather than low power delivery (35 W) over a long duration. These advances could further decrease the incidence of cardiac perforation.

The incidence of PPIS seems strongly to age, gender, and underlying disease-related. Some studies suggest that elder individuals were more prone to develop cardiac perforation-related PPIS, which is probably because aging may be associated with an increased inflammatory response (39). Multivariate analysis revealed that the incidence of acute pericarditis post-ablation in females increased by 40% compared with that of male patients. One possible explanation for this is that as the thickness of the left atrial wall decrease, so too does the increased risk of pericardial compilations in females, especially in patients with AF (40). Additionally, the risk of developing acute pericarditis was 40% higher in obese patients (41). Pleural incision, anemia, and rheumatoid arthritis were also identified as independent risk factors (10, 42). In an attempt to preclude the onset of this complication, high-risk individuals should have priority to be paid close attention.

Diagnosis depends upon clinical suspicion and the exclusion of other clinical conditions that may mimic this syndrome, such as pulmonary embolism, pneumonia, and congestive heart failure. Transthoracic echocardiography is a readily available imaging modality performed at the bedside to assess cardiac anatomy, function, and hemodynamics. It is the golden standard in the determination of accurate diagnosis and the tool of choice for emergent bedside evaluation for cardiac tamponade (43). In practice, the fluoroscopic check of cardiac motion provides a useful tool for the early recognition of pleural effusion. CT can provide information on pericardial thickening, calcification, effusions, and lead perforations. Badger et al. found that quantification of left atrial structural remodeling with delayed-enhancement magnetic resonance imaging has been overestimated in patients undergoing RFCA in the acute stage because of an inflammatory process induced by radiofrequency energies (44). Therefore, both pericardial and pleural effusions typically result from thermal injury. Additionally, routine laboratory tests and radiographic investigations usually show non-abnormalities in patients with PPIS, such as leukocyturia, pleural effusion, or pericardial effusion. Treatment strategies for PPIS aim at decreasing pericardial inflammation and improving symptoms. Based on the European Society of Cardiology 2015 guidelines, nonsteroid anti-inflammatory agents (NSAIDs) and colchicine are the preferred drugs for the treatment of PPIS. Aspirin has become the first choice for NSAIDs because of its analgesic and anti-inflammatory effects. Steroids are second-line agents for symptom control, which should be tapered when inflammatory markers are normalized and clinically significant symptoms are reduced (28, 45, 46). Colchicine-resistant patients or steroid-dependent patients should choose other therapeutic options such as anakinra or intravenous immunoglobulins (47). The combination of colchicine and NSAIDs in the treatment of PPIS has been reported to be associated with lower rates of pericardiocentesis and reduced clinically significant symptoms (28, 48, 49).

Typically, the treatment of PPIS was well-received, and the results are promising, but hospital stay may be prolonged, and healthcare costs may be increased. Initial investigations of clinically suspected PPIS were serum inflammatory levels and echocardiography. When the results of those tests are inconclusive, computerized tomography can provide additional diagnostic information. The diagnosis of PPIS diagnosis remains difficult in cases following cardiac catheter intervention because patients had no clinical signs of manifest heart disease. Therefore, cardiologists and pulmonologists should be aware of this rare but potentially important complication (50).

## Conclusion

We report a rare case of PPIS that is characterized as massive pleural effusion alone after RFCA of AF. To reduce potential progression, timely diagnostics and preventive strategies for PPIS after RFCA of AF are of great importance. PPIS should be considered in a patient who presents with massive pleural effusion alone following RFCA, especially after an infectious cause and pulmonary embolism have been excluded.

## Data availability statement

The original contributions presented in the study are included in the article/supplementary material, further inquiries can be directed to the corresponding authors.

## Ethics statement

Written informed consent was obtained from the individual(s) for the publication of any potentially identifiable images or data included in this article.

## Author contributions

YW and GC performed the operation and revised the study. MH drafted the manuscript. GC and YB organized the study and edited the manuscript. All authors have read and agreed to the published version of the manuscript.

## Funding

This study was supported by grants from Tongji Hospital Returns from Studying Abroad Startup Foundation (2022hgry008 and 2022hgry023).

## Conflict of interest

The authors declare that the research was conducted in the absence of any commercial or financial relationships that could be construed as a potential conflict of interest.

## Publisher's note

All claims expressed in this article are solely those of the authors and do not necessarily represent those of their affiliated organizations, or those of the publisher, the editors and the reviewers. Any product that may be evaluated in this article, or claim that may be made by its manufacturer, is not guaranteed or endorsed by the publisher.
